# Sustainable Adhesive Formulation and Performance Evaluation of Bacterial Nanocellulose and Aloe Vera for Packaging Applications

**DOI:** 10.3390/molecules30153136

**Published:** 2025-07-26

**Authors:** Urška Vrabič-Brodnjak, Aljana Vidmar

**Affiliations:** Faculty of Natural Sciences and Engineering, Department of Textiles, Graphic Arts and Design, University of Ljubljana, Aškerčeva 12, 1000 Ljubljana, Slovenia; aljanavidmar@gmail.com

**Keywords:** bio-based adhesives, packaging applications, sustainable materials, synthetic adhesive alternatives, substrate interaction

## Abstract

The development of bio-based adhesives as sustainable alternatives to synthetic formulations presents a significant opportunity for advancing environmental sustainability in packaging applications. This research aimed to develop and evaluate a bio-based adhesive derived from bacterial nanocellulose (BNC), aloe vera and its mixtures as a potential replacement for commercial synthetic adhesives. Aloe vera, selected for its polysaccharide-rich composition, served as a natural polymeric matrix, while BNC contributed reinforcing properties. The adhesive formulations, with and without BNC, were compared to a commercial adhesive to assess their mechanical performance. T-peel and shear tests were conducted on smooth and rough paper substrates to evaluate adhesive strength. The bio-based adhesive incorporating BNC demonstrated superior shear and peel strength on rough substrates due to enhanced mechanical interlocking within the fibrous structure of paper, whereas performance on smooth surfaces was hindered by uneven BNC distribution, reducing adhesive-substrate interaction. Although the commercial adhesive achieved higher absolute maximum force values, the bio-based formulation exhibited comparable mechanical stability under specific conditions. These findings underscore the influence of substrate properties and application methods on adhesive performance, highlighting the potential of bio-based adhesives in packaging applications and the need for further formulation optimization to fully realize their advantages over traditional synthetic adhesives.

## 1. Introduction

Adhesives are indispensable materials that underpin a vast array of industrial applications by enabling the formation of reliable, durable joints critical to product functionality, structural integrity, and longevity. Among these applications, the packaging sector is one of the largest consumers of adhesives, demanding materials that not only exhibit exceptional mechanical robustness but also meet increasingly stringent environmental and sustainability criteria [1,2,3]. Historically, synthetic adhesives—predominantly petrochemical-derived polymers—have dominated this sector due to their superior adhesion performance, ease of processing, and cost-effectiveness. However, these materials pose significant environmental concerns related to their non-renewable origin, resistance to biodegradation, and potential ecological toxicity upon disposal or incineration, contributing to pollution and resource depletion. The urgent global imperative to mitigate environmental impact and promote circular economy principles has catalyzed a paradigm shift toward the development of sustainable, bio-based adhesive alternatives [4,5,6]. Renewable polymers derived from biomass offer promising pathways to reduce reliance on fossil fuels and decrease the carbon footprint of adhesive formulations. Within this context, bacterial nanocellulose (BNC), a highly pure microbial polysaccharide synthesized extracellularly by bacteria such as *Gluconacetobacter xylinus*, has emerged as an exceptional candidate for bio-adhesive reinforcement [7,8,9,10]. BNC is characterized by its nanoscale fibrillar network, which imparts remarkable tensile strength, high crystallinity, flexibility, and biodegradability. Furthermore, its capacity to form strong hydrogen-bonded networks enhances interfacial adhesion within polymeric matrices, thereby improving the mechanical and functional properties of bio-based adhesives [9,10,11,12].

Complementing BNC, aloe vera (*Aloe barbadensis Miller*) is a succulent plant whose mucilaginous gel is rich in bioactive polysaccharides, which exhibit intrinsic adhesive and film-forming properties [12,13]. The gel’s viscous nature and biocompatibility make aloe vera a valuable natural polymeric matrix for adhesive systems. The strategic integration of BNC and aloe vera in adhesive formulations leverages the synergistic interplay between nanofibrillar reinforcement and polysaccharide adhesion, potentially yielding bio-based adhesives with competitive performance metrics relative to their synthetic counterparts [14,15,16,17,18].

The goal of this study was to develop and systematically characterize a bio-based adhesive formulated from BNC and aloe vera, aiming to deliver mechanical performance and adhesion properties comparable to commercial synthetic adhesives while offering enhanced environmental sustainability. The research focused on optimizing adhesive formulations, evaluating their mechanical and interfacial properties on diverse substrates, and assessing their potential as sustainable alternatives for industrial applications, particularly in the packaging sector.

Adhesive performance was systematically evaluated against a benchmark commercial synthetic adhesive through mechanical testing on substrates varying in surface roughness and physicochemical properties. This research directly addresses the rising regulatory and market-driven mandates—embodied by policies such as the European Green Deal—that promote sustainable material usage, reduction of environmental impact, and advancement of circular economy strategies. The principal objectives of this work were as follows: (1) to conduct an exhaustive market and literature review of adhesive technologies relevant to packaging; (2) to optimize the cost-efficient biosynthesis of BNC using agro-industrial by-products and alternative nutrient sources; (3) to develop and optimize aloe vera–BNC composite adhesive formulations; (4) to perform mechanical and adhesion strength testing on relevant substrates; and (5) to benchmark these bio-based adhesives against commercially available synthetic adhesives to assess their industrial viability.

The literature demonstrates that cellulose nanocrystals (CNCs), closely related to BNC, enhance the mechanical and adhesive properties of pressure-sensitive adhesives (PSAs) [12,14,18]. Reinforcement is most pronounced when CNCs are incorporated via in situ polymerization, facilitating homogeneous nanocrystal dispersion and strong interfacial bonding within the polymer matrix. Similarly, nanocellulose fillers synthesized through suspension polymerization methods have been shown to increase adhesive strength, peel resistance, and glass transition temperature, alongside elevated storage modulus values indicative of improved cohesive integrity. Moreover, the inclusion of nanocellulose in wood adhesives has been reported to enhance mechanical properties, dimensional stability, and simultaneously reduce harmful formaldehyde emissions from synthetic adhesives, highlighting its multifunctional benefits. Despite these advances, challenges remain in scaling production and optimizing processing techniques for industrial deployment.

Additional investigations into the use of nanocellulose as an additive to hydroxypropyl cellulose (Klucel E) formulations reveal increased penetration into wood substrates, improved retention, and enhanced compressive strength of treated materials [16]. These treatments also exhibit reduced discoloration and superior resistance to environmental aging, indicating potential for long-term durability improvements. In the cosmetic industry, bacterial nanocellulose has gained prominence as a biodegradable and renewable alternative to synthetic polymers such as nylon and polyethylene glycol derivatives. Its efficacy as both a carrier for active ingredients and a structuring agent enhances formulation stability, texture, and bioavailability, with emerging applications in packaging, cosmetic, wood industry and more [17,18,19,20]. In the field of food packaging, nanocellulose incorporation enhances barrier properties against oxygen and microbial contamination, thereby contributing to extended product shelf life. However, due to its hydrophilic nature, nanocellulose does not inherently improve moisture barrier properties unless it is chemically modified or combined with hydrophobic materials. Additionally, nanocellulose enables the development of biodegradable adhesives with active preservation functionalities, such as antioxidant or antimicrobial additives [19,20,21,22,23]. Complementary studies focusing on aloe vera-based biopolymer films have confirmed the polysaccharide’s capacity to enhance tensile strength, UV resistance, moisture barrier properties, and biodegradability [24,25,26,27,28]

These attributes substantiate aloe vera’s role as a functional component within bio-based adhesives rather than a passive matrix [13,15]. Preliminary data also suggest that aloe vera’s adhesive strength can be augmented via chemical or physical modification, though comprehensive studies on durability, aging, and scalability are still needed.

The extant research underscores the considerable synergistic potential of combining bacterial nanocellulose and aloe vera to develop bio-based adhesives that meet the dual imperatives of mechanical performance and sustainability. Nonetheless, several challenges must overcome to realize their industrial applicability. These include the high production costs of nanocellulose materials, technological hurdles in large-scale processing, and incomplete toxicological and environmental impact assessments [29]. Addressing these issues through multidisciplinary research is vital to optimize nanocellulose integration, improve adhesive formulation stability, and enable commercial-scale production of eco-friendly adhesives. The advancement of bio-based adhesive technologies based on bacterial nanocellulose and aloe vera presents a promising avenue to reduce dependency on synthetic adhesives, facilitate biodegradability, and foster sustainable innovation in packaging and other industrial sectors. This research contributes to the broader scientific effort to develop materials that reconcile high performance with environmental stewardship, supporting the transition toward a more sustainable materials economy.

## 2. Results

### 2.1. Results of Basic Properties of the Cardboard

The structural properties of the tested cardboard samples were characterized by analyzing grammage, thickness, specific volume, and density, all of which are interrelated and influenced by the molecular organization of the fibrous network (Table 1). No one-way ANOVA was performed for these measurements, as only basic physical parameters of a single cardboard type were evaluated. These values (grammage, thickness, density, and specific volume) serve as descriptive baseline characteristics and do not involve comparisons across multiple groups or treatments. Therefore, no meaningful statistical correlation or further analysis with adhesive performance could be drawn from this dataset.

Grammage reflects the amount of fibrous material present and is directly associated with the concentration of cellulose microfibrils and their distribution across the sheet. Samples with higher grammage generally exhibited increased thickness, attributed to a more voluminous fiber network and reduced fiber consolidation. Specific volume provides insight into the porosity and spatial arrangement of fibers; higher specific volume indicates a looser, more open structure with greater inter-fiber voids. Conversely, density structures are indicative of stronger intermolecular hydrogen bonding and lower air content within the matrix, which can enhance mechanical strength, surface smoothness, and barrier properties. These properties at the microstructural level are critical for determining the functional performance of cardboard in packaging applications, influencing rigidity, printability, and resistance to moisture and deformation, which is important also for the adhesive applications.

### 2.2. Structural Properties and Water Absorbency of the Cardboard

Porosity of the cardboard is the capacity of a fibrous material to permit penetration of liquids and gases and presents a key factor influencing the performance of paper-based substrates, particularly in packaging and adhesive applications. The intrinsic porosity of paper arises from the interconnected network of air voids within the fiber matrix, which affects not only fluid absorption but also mechanical strength, surface topography, and ultimately adhesion quality. In this study, porosity and surface roughness were analyzed on both the coated and uncoated sides of cardboard samples. The results have shown that the rough, uncoated surface exhibited an airflow rate 8.7 times higher than the smooth, coated side, reflecting significant differences in fiber packing and surface morphology (Table 2). The coated side showed greater measurement variability due to airflow values nearing the detection limits of the instrument. Bendtsen smoothness was used as an indirect measure of surface roughness, quantifying air passage through the material under standardized conditions. Higher airflow indicates greater surface roughness and lower resistance to gas penetration, providing complementary insight into porosity and surface characteristics. The coated surface demonstrated extremely low porosity (<5 mL/min), indicative of a highly compact and sealed fiber network where the coating fills inter-fiber voids and limits capillary pathways at the molecular level. In contrast, the uncoated side showed significantly higher porosity (51.2 mL/min), consistent with a more open fiber structure and accessible voids that facilitate gas permeability. The reliability of measurements on the uncoated surface was confirmed by a coefficient of variation of 5.85%, while variability on the coated side could not be reliably determined due to instrumental limits.

These structural differences profoundly impact adhesive performance. The low porosity and smoothness of the coated surface reduce adhesive penetration and mechanical interlocking, potentially limiting adhesion strength unless compatible adhesives or surface treatments are employed. Conversely, the higher porosity and roughness of the uncoated side enhance adhesive wetting and penetration, promoting stronger mechanical bonding through increased surface area and micro-scale anchoring sites.

The water absorbency values (Cobb) for the cardboard samples, indicating an average of 16.62 g/m^2^ for the coated side, classifying it as fully bonded. In contrast, the rough, uncoated side exhibited a higher average water absorption of 21.00 g/m^2^, corresponding to a half-bonded classification. The low standard deviations (0.55 g/m^2^ for the coated side and 0.07 g/m^2^ for the uncoated side) demonstrate the precision and reliability of the measurements.

These findings confirm that the smooth, coated surface possesses stronger fiber bonding and enhanced hydrophobicity, whereas the rough, uncoated surface exhibits weaker bonding and increased hydrophilicity, consistent with the observed structural and surface characteristics of the samples. Table 3 presents the *p*-values obtained from the ANOVA analysis.

### 2.3. Results of the Viscosities of the Used Adhesive Mixtures

Table 4 presents the results of the used adhesive mixtures that were later used for the cardboard bonding. According to the results, pure aloe vera gel exhibited a viscosity of 10.0 Pa·s, indicative of its polysaccharide-rich composition and extensive hydrogen bonding between polymer chains. Upon incorporation of bacterial nanocellulose, the viscosity increased to 16.2 Pa·s, suggesting that viscosity of the aloe vera + BNC sample compared to pure aloe vera is due to the entangled nanofibrillar network of BNC, which reinforces the gel structure. BNC’s high aspect ratio and water-holding capacity enhance the internal network, restricting flow and increasing viscosity. Additionally, possible interactions between aloe vera polysaccharides and BNC fibers may further stabilize the system. The commercially based adhesive demonstrated a higher baseline viscosity of 19.9 Pa·s, consistent with its densely crosslinked polymer matrix. The addition of BNC further increased the viscosity to 24.7 Pa·s, likely due to the nanocellulose’s high aspect ratio and strong intermolecular hydrogen bonding capacity, which enhance physical entanglement and network reinforcement within the adhesive matrix. These results underscore the complex role of BNC as both a rheological modifier and a structural reinforcement agent, with effects highly dependent on the molecular compatibility and interaction dynamics of the base material.

### 2.4. Results of Mechanical Characterization of Cardboard Bonded with Various Adhesives

#### 2.4.1. Results of Shear Strength Test of Adhesive Joint

(a) Results of Shear Strength Test of Aloe Vera with and without BNC

Shear strength testing of aloe vera adhesive on coated and uncoated cardboard surfaces revealed significant differences in mechanical performance, influenced by both surface type and the presence of bacterial nanocellulose.

**Figure 1:**
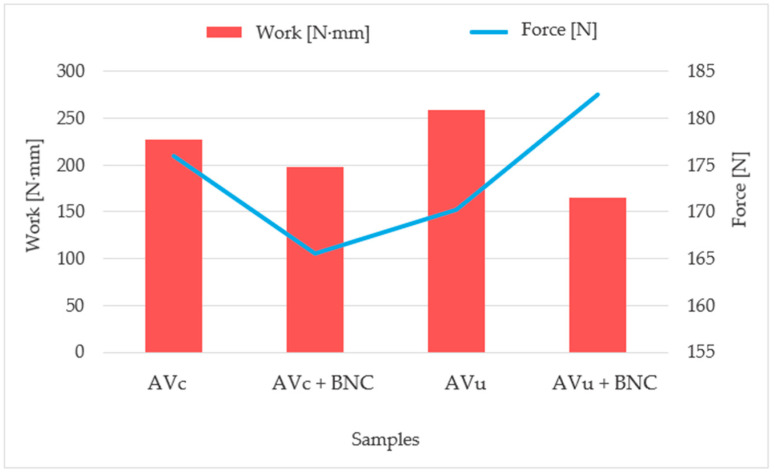
Results of work and average maximum shear force for aloe vera adhesive with and without BNC, on carboard surfaces (uncoated and coated).

As shown in Figure 1, BNC addition significantly affected maximum force and average work values. On the uncoated surface, BNC increased the average maximum force from 170.18 N to 182.50 N, likely due to improved mechanical anchoring within the fibrous structure. However, average work decreased from 258.44 N∙mm to 164.72 N∙mm, indicating reduced elasticity and higher joint stiffness. On the coated side, BNC addition led to a decrease in maximum force (from 175.97 N to 165.51 N) and average work (from 227.42 N∙mm to 198.15 N∙mm). This may result from uneven adhesive distribution caused by BNC, reducing contact and joint performance. As shown in Figure 1, the effect of BNC on mechanical properties is surface-dependent, improving strength on rough substrates but reducing performance on smooth ones. In applications requiring enhanced shear strength on uncoated (rough) surfaces, the incorporation of BNC is recommended due to its contribution to improved mechanical anchoring. Conversely, for bonding to coated (smooth) surfaces, the use of adhesive formulations without BNC is more effective, as they exhibit superior interfacial adhesion and higher shear strength.

(b) Results of Shear Strength test of Commercial Adhesive with and without BNC

**Figure 2:**
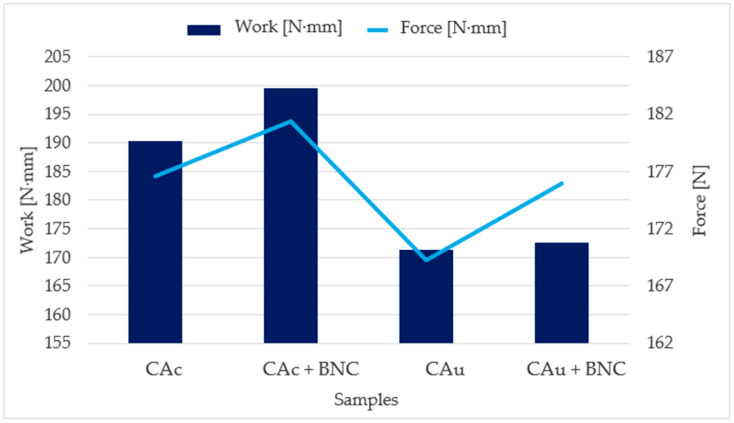
Results of work of rupture and average maximum shear force for commercial adhesive with and without BNC, on carboard surfaces (uncoated and coated).

In shear strength testing of commercial adhesive and BNC, the samples exhibited relatively high maximum forces, as illustrated in Figure 2.

Variations in the peak forces achieved with the commercial adhesive were minimal, with differences not exceeding 8%. This low variability is attributed to cohesive failure within the cardboard substrate rather than at the adhesive interface, indicating that rupture consistently occurred within the fibrous layer. Consequently, the results demonstrated high repeatability, reflecting the robustness and reliability of the commercial adhesive formulation. Statistical analysis of maximum force values revealed no significant differences between coated and uncoated sides of the cardboard, regardless of BNC incorporation. Thus, it cannot be concluded that BNC addition enhances shear strength in terms of peak force. However, more notable effects were observed in the analysis of the work performed up to maximum force, which provides insight into the energy absorption capacity of the adhesive joints. In these cases, the inclusion of BNC contributed to increased energy dissipation under shear loading, suggesting improved ability of the joint to absorb and distribute mechanical stress.

#### 2.4.2. Results of T-Peel Test of Adhesive Joint

The T-peel test demonstrated reliability as a method and confirmed that the incorporation of BNC enhances the mechanical performance of the adhesive joint. The results, presented in followed subchapters, clearly indicate an increase in the average maximum force when aloe vera with BNC and commercial adhesive with BNC were applied—both on the coated and uncoated sides of the cardboard.

(a) Results of T-Peel test of Aloe Vera with and without BNC Applied on the Cardboard.

When aloe vera was used as an adhesive on the rough side of the cardboard without the addition of BNC, low maximum force values were recorded (0.77 N). This low bond strength, accompanied by high variability, is primarily attributed to the high-water content of aloe vera. Although it wets the surface effectively, aloe vera leaves only a minimal amount of solid residue after drying, which is insufficient to form a robust adhesive layer or to ensure a uniform distribution of solids. With the incorporation of BNC, the average maximum force increased to 1.19 N, representing a 35.4% improvement. This enhancement suggests a more homogeneous adhesive distribution and improved bonding performance; however, the adhesive strength remained inadequate for effective fiber bonding, as also presented in Table 5.

On the coated side of the cardboard, aloe vera without BNC achieved significantly higher maximum forces (3.35 N). This superior performance is attributed to reduced absorption of the adhesive into the surface, allowing a greater amount of adhesive to remain at the interface, thereby strengthening both cohesive and adhesive interactions. The addition of BNC led to a modest further increase in maximum force, likely due to improved cohesive properties. Despite this, the adhesive joints did not reach the threshold required for permanent bonding or fiber tearing. Notably, although the BNC-enriched adhesive exhibited higher short-term bonding strength, the joints weakened rapidly after reaching the maximum force and failed by detaching in larger fragments. To further assess the ductility or elasticity of the adhesive joints, the work of rupture by the adhesive up to the average maximum force was calculated using numerical integration via the trapezoidal method. The data show that on the smooth side, the addition of BNC significantly enhanced bonding performance, increasing the maximum force by 35% and the work of rupture by 56.5%. This indicates that the joint became more ductile and capable of absorbing greater energy. In contrast, on the uncoated side, the addition of BNC resulted in a 90% increase in maximum force but a 13% decrease in the work of rupture. This suggests the formation of a stiffer but less energy-absorbing joint.

The one-way ANOVA test was conducted to evaluate whether the differences in force and work of rupture among the four samples (AVc, AVc + BNC, AVu, AVu + BNC) and showed statistical significance (Table 6). The *p*-values obtained for both properties were below the significance level of 0.05 (*p* < 0.001), indicating strong evidence that at least one group differs significantly from the others. This confirms that the addition of BNC and the type of sample (coated vs. uncoated) significantly affect the mechanical properties measured.

(b) Results of T-Peel Test of Commercial Adhesive with and without BNC Applied on the Cardboard.

As anticipated, adhesive joints formed with the commercial adhesive exhibited higher average maximum forces than those bonded with aloe vera on both the coated and uncoated sides of the cardboard. The results presented in Table 7 further demonstrate that the incorporation of BNC enhanced the average maximum force values on both surfaces.

On the coated side of the board, the average maximum force for pure commercial adhesive was 9.781 N, with a coefficient of variation of 20.39%. The addition of bacterial nanocellulose (BNC) increased the maximum force to 11.041 N, accompanied by a slightly higher variation of 23.73%. On the uncoated side, the measured forces were somewhat lower; pure commercial adhesive achieved an average maximum force of 9.457 N with a variation of 25.13%, while the incorporation of BNC raised the force to 10.558 N, though with an increased variation of 27.36%. The results suggest that the addition of BNC enhances the mechanical strength of the adhesive joint on the coated side of the board. Therefore, it cannot be conclusively stated that BNC contributes to a stronger adhesive bond, as the observed differences may arise from inherent variability. Despite the increased variation observed with BNC addition, the results are promising and suggest potential for further optimization of the adhesive formulation.

The one-way ANOVA test was performed to evaluate whether the means of force and work of rupture differed significantly among the commercial adhesive samples with and without BNC additive, applied to coated and uncoated cardboard surfaces. (Table 8).

### 2.5. Results of SEM Analysis of Cardboard with Different Adhesives Applied

Scanning electron microscopy was used to examine the uncoated sides of cardboard bonded with aloe vera adhesive, with and without bacterial nanocellulose.

**Figure 3:**
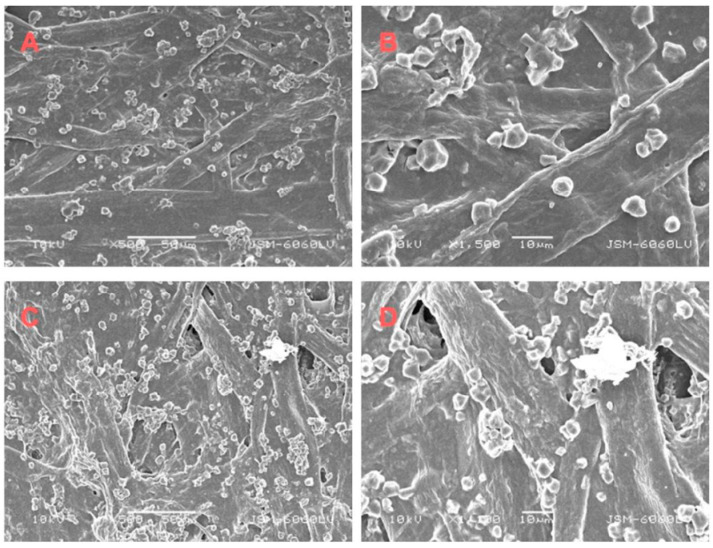
SEM images of an aloe vera sample on the uncoated side of the cardboard, without BNC (**A**,**B**) at magnifications of 500× and 1500× and with BNC (**C**,**D**) at magnifications of 500× and 1100×.

Without BNC, the adhesive penetrated the fibrous structure effectively, forming hydrogen bonds and physical interlocks that ensured stable mechanical anchoring (Figure 3). However, incomplete filling of deeper voids limited maximum load capacity. With BNC addition, a denser adhesive layer was observed. Although individual BNC particles were not visible—likely due to their nanoscale size and integration into the matrix—their presence enhanced mechanical anchoring via increased hydrogen bonding and network formation. However, local BNC agglomerates caused stress concentrations, reducing elasticity and energy absorption. These results indicate that while BNC improves adhesive performance, uniform dispersion is critical to avoid negative effects on joint flexibility.

**Figure 4:**
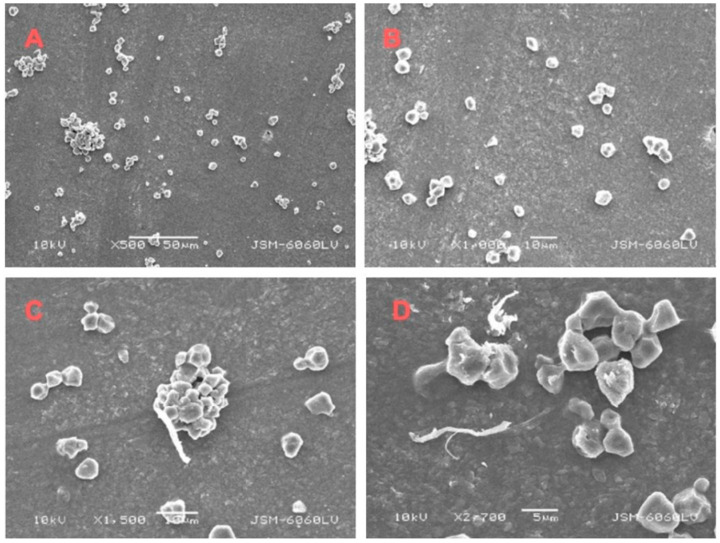
SEM images of an aloe vera sample on the coated side of cardboard, without BNC (**A**,**B**) at magnifications of 500× and 1000× and with BNC (**C**,**D**) at magnifications of 1500× and 2700×.

SEM analysis of the coated side of the cardboard treated with aloe vera adhesive without BNC addition revealed an inhomogeneous and limited adhesive distribution across the smooth, coated surface (Figure 4). At 500× magnification, discrete adhesive aggregates were observed, indicating incomplete surface coverage and poor wetting, which suggest weak physicochemical interactions between the adhesive and the low-porosity coated substrate. Higher magnification (1000×) further confirmed the presence of fine, dispersed particles lacking continuity, implying a discontinuous adhesive film that may compromise bond strength. Given that the coated surface does not support mechanical interlocking, adhesion in this case relies primarily on surface forces such as van der Waals interactions and hydrogen bonding. However, the limited contact area likely weakens these interactions. The addition of BNC was not detectable in SEM images, likely due to its nanoscale dimensions, low concentration, minimal layer thickness, or the inherent smoothness of the coated surface reducing topographic contrast. Only cellulose-like particles were visible, which may originate from either the cardboard substrate or aloe vera components.

**Figure 5:**
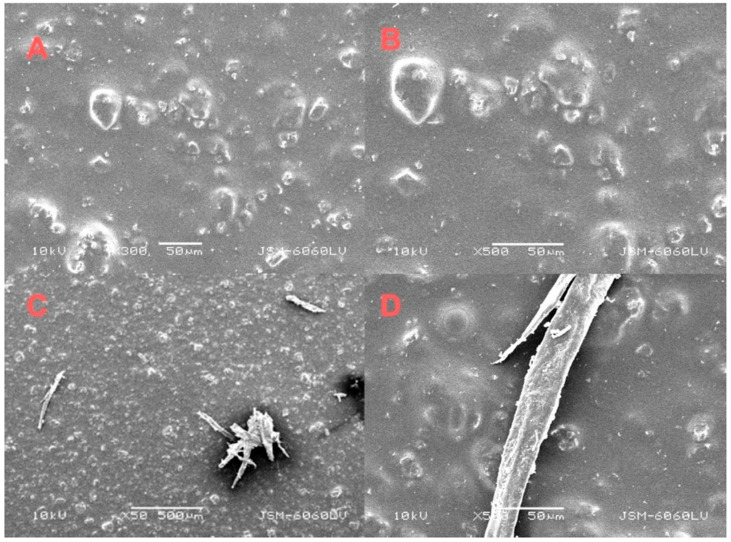
SEM images of the commercial adhesive on a sample on the coated side of a cardboard, without BNC (**A**,**B**) at magnifications of 300× and 500× and with BNC (**C**,**D**) at magnifications of 50× and 500×.

SEM analysis of the commercial adhesive applied to the coated side of the cardboard at magnifications of 300× and 500× revealed a uniform and continuous distribution across the surface, with no significant local accumulations observed (Figure 5). This homogeneity is consistent with the higher maximum force and average work values recorded in mechanical testing, indicating effective load transfer and cohesive bonding. The addition of bacterial nanocellulose was not detectable in the SEM images, likely due to its nanoscale dimensions and integration into the adhesive matrix. However, occasional cellulose particles were visible, which may be attributed to mechanical damage or fiber exposure caused by the cutting of the cardboard substrate.

**Figure 6:**
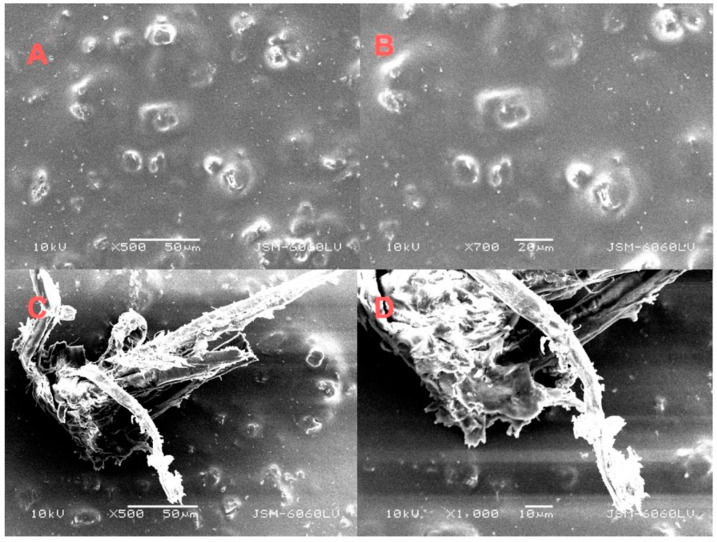
SEM images of the commercial adhesive on a sample on the uncoated side of the cardboard, without BNC (**A**,**B**) at magnifications of 500× and 700× and with BNC (**C**,**D**) at magnifications of 500× and 1000×.

Based on SEM analysis of the commercial adhesive with and without the addition of BNC on the uncoated side of the cardboard, conducted at magnifications of 500× and 700× (Figure 6), a uniform adhesive layer was observed to be well mechanically anchored within the fibrous network. The surface was evenly coated, with no visible aggregates or irregularities, enabling strong adhesion and uniform load distribution across the interface. The homogeneous distribution of the adhesive contributed to enhanced joint elasticity by preventing localized stress concentrations, thereby allowing effective deformation transfer and improving the overall mechanical performance of the bond. The presence of BNC was not detected in the SEM images, likely due to its nanoscale size or integration within the adhesive matrix. Only fragments resembling cellulose were observed, which are presumed to result from mechanical disruption during the cutting of the cardboard specimens.

## 3. Materials and Methods

### 3.1. Materials and Adhesive Preparation Processes

In this study, the adhesive strength of different adhesive formulations was investigated. Aloe vera was used as the natural base for the experimental adhesive, and BNC was added with the goal to enhance its adhesive properties. For comparison, the commercial adhesive was also modified with the addition of BNC to assess its effect on joint strength. It was hypothesized that the incorporation of BNC would lead to improved mechanical properties in both adhesives, resulting in increased adhesive joint strength.

For adhesive mixtures, different materials were used such as the following:

1. Commercial, dispersion adhesive, which is a mixture of polymer dispersion, additives and water (Mitol d.o.o., Sežana, Slovenia).
2. Aloe vera gel at 99% pure (*Aloe barbadensis miller*), (Aloe Labs, inc., Harlingen, TX, USA)
3. Bacterial nanocellulose (BNC), produced in the Pulp and Paper Institute, Ljubljana Slovenia, as described Lavrič et al. [14]

To analyze and test the properties of the adhesives and their applications, one-side coated white cardboard (certified properties: grammage 250 g/m^2^, thickness 0,3 mm) was used as base material.

The preparation steps of BNC and adhesive mixtures are presented in the (a) and (b) subchapters.

(a) Preparation of BNC for adhesive applications.

Before mixing BNC into other adhesive solutions, the following procedure was carried out. A 100 mL liquid BNC sample with an 80% water content was measured into a measuring cylinder, previously washed with distilled water. Due to the high-water content, it was not mixed directly with aloe vera, as this would have caused excessive dilution of the mixture. Instead, the BNC was pre-soaked to reduce the water content. The soaked sample was further dried in a drying oven at 57 °C for 60 min, avoiding complete dehydration to facilitate homogeneous mixing with both commercial adhesive and aloe vera.

(b) Incorporation of BNC into the adhesive matrix

For optimal homogenization, a Wisestir 30D mixer (Millot Oy Science, Porvoo, Finland) was employed, capable of processing materials with viscosities up to 10,000 mPas and allowing adjustment of rotational speed from 200 to 3000 rpm. A volume of 100 mL of the commercial adhesive (corresponding to a mass of 120 g) was measured using a graduated cylinder. Subsequently, 2 g of BNC was added, and the mixture was stirred for 30 min under controlled conditions: 10 min at 600 rpm, 10 min at 700 rpm, and 10 min at 800 rpm.

An identical procedure was applied to the aloe vera-based adhesive. Specifically, 100 mL of aloe vera (mass 90 g) was combined with 2 g of BNC and mixed under the same conditions as described above. Following mixing, the aloe vera−BNC dispersion was allowed to rest to enable partial bubble release, as the presence of entrapped air could negatively impact the adhesive bond quality. Complete degassing was not achieved, and no anti-foaming agents were utilized.

As part of the experimental work, four adhesive formulations were used and prepared:

4. Pure aloe vera (100 mL) (Sample name: AV);
5. Pure commercial adhesive 1001 (Sample name: 100 mL) (CA);
6. 100 mL of aloe vera with the addition of 2 g of BNC (sample name: AV + BNC);
7. 100 mL of commercial adhesive with the addition of 2 g of BNC (sample name: CA + BNC).

Adhesive mixtures and mixing conditions are presented in Table 9.

Combinations of adhesives and applications to the carton, that had one side coated and one side uncoated surfaces, are presented in Table 10. The bonding length on the carton was 37 mm, and a 1 kg load was placed on top of each glued sample for 1 h.

### 3.2. Methods

#### 3.2.1. Analysis of the Cardboard

(a) Basic, structural properties of the cardboard

The basic properties of the cardboard, which influence other material characteristics discussed in subsequent sections, were required to be determined. The grammage of all samples was determined in accordance with ISO 536:2019 [30]. Ten specimens from each paper type were cut to dimensions of 10 × 10 cm and subsequently weighed. The thickness of the samples was measured using a digital micrometer (Mitutoyo Corp., Kanagawa, Japan) with a precision of 0.001 µm at ten randomly selected locations on each sheet, following the procedure outlined in ISO 534:2011 [31]. From the grammage and thickness, the density and specific volume of each sample paper were calculated, as described in the ISO 534 standard. All carboard samples were conditioned and analyzed at a temperature of 23 °C and a relative humidity of 50%.

(b) Surface properties and water absorption capacity (Cobb method) of the cardboard

Since paper consists of a randomly matted network of fibers, its structure exhibits varying degrees of porosity. To assess the bonding efficiency, the surface properties of the paper were analyzed. Porosity, smoothness, and Cobb value measurements were conducted on the original cardboard substrate prior to adhesive application. The term “coated side” refers to the manufacturer-applied surface treatment, typically consisting of a synthetic or mineral-based coating designed to enhance printability and moisture resistance. This industrial coating is applied during the cardboard manufacturing process and is not related to the adhesive formulations used in this study. The measurements of smoothness and porosity were carried out using a Bendtsen instrument (Alat UJI, Jakarta, Indonesia). The measurement range was 0 to 5000 mL/min, with air flowing through a flat metal ring and the test sample. The pressure difference was recorded using the rotameter tube provided. Smoothness and porosity were measured on both sides of the specimen and ten replicates were performed at each test.

Water absorption capacity was quantified using the Cobb60 method (g/m^2^) in accordance with ISO 535 [32]. In this procedure, 100 mL of water was applied to the cardboard surface for 45 s, after which the increase in mass was measured. Ten replicate measurements were performed for the cardboard and each side (coated and uncoated).

#### 3.2.2. Analysis of the Viscosity of the Adhesives

The viscosity of the adhesives was measured in accordance with EN 12092:2002 [33] using a rotational viscometer (Brookfield, Anton Paar GmbH, Graz, Austria). The measurements were carried out with a stainless steel spindle at a rotational speed of 1.5 rpm. As the drying time is strongly influenced by the temperature, the two parameters were analyzed together.

#### 3.2.3. Mechanical Characterization of Cardboard Bonded with Various Adhesives

(a) Shear Strength Test

To evaluate the shear strength of the adhesives, 40 test specimens were prepared from cardboard with a grammage of 250 g/m^2^, each measuring 100 mm in length and 25 mm in width. Four different adhesive formulations were used: aloe vera, aloe vera with BNC, commercial adhesive, and commercial adhesive with BNC. For each adhesive formulation, 10 specimens were bonded using the coated surfaces and 10 using the uncoated surfaces. Consequently, for each adhesive type, 10 specimens were allocated for shear strength testing—five with the coated sides adhered and five with the uncoated sides adhered. The adhesive was applied using a coater machine (RKPrint coater, Herts, United Kingdom), assisted by a transparent binding film and a writing pad to ensure uniform distribution. Each specimen was coated with adhesive over 37 mm. Following the application, the bonded specimens were subjected to a constant load by placing a 2.5 kg package of office paper on top. The specimens remained under this load for 20 h to allow proper adhesion and curing.

(b) T-Peel Test

To determine the peel strength by means of the T-Peel test, 40 test specimens were prepared from cardboard with a grammage of 250 g/m^2^. The specimens were cut to dimensions of 120 mm in length and 25 mm in width. Adhesives were applied to a 40 mm section of each specimen using a spreading machine to ensure uniform and consistent distribution. After bonding, a weight of 2.5 kg was placed on top of the specimens, which were then left to dry under this load for 20 h at room temperature (RH 5%; 23 °C). For each adhesive formulation, 10 test specimens were prepared, five with the coated sides adhered together and five with the uncoated sides adhered. To evaluate the shear strength of the adhesive joints and to perform the T-Peel test, a universal testing machine dynamometer (Instron 5567, Darmstadt, Germany) was employed. This device is designed for measuring tensile properties of a wide range of materials, including textiles, paper, cardboard, and polymer-based substrates, in accordance with ISO 2062:1997 [34].

As part of the measurement of peel and shear forces, displacement data were also recorded. These data enabled the calculation of the total work performed up to the point of maximum force, thereby allowing an assessment of the brittleness or ductility of the adhesive joint. Since work corresponds to the integral of force with respect to displacement, the trapezoidal numerical integration method was employed. This method is commonly used for calculating work in cases where discrete data points for force and displacement are available.

#### 3.2.4. Scanning Electron Microscopy (SEM) Analysis of Cardboard Surfaces with Applied Adhesives

The surface was also analyzed using a scanning electron microscope (SEM). Prior to imaging, the samples were sputter-coated with a thin layer of gold, to enhance conductivity for electron microscopy. Micrographs of sample surfaces were taken with a scanning electron microscope JSM6060 LV (Jeol Ltd., Tokyo, Japan). The instrument operated at 10 kV and at different magnifications.

#### 3.2.5. Statistical Analysis

Two adhesive types (aloe vera-based and commercial polyvinyl acetate) were tested on coated and uncoated cardboard surfaces, with and without 2 g BNC per 100 mL adhesive. This yielded four sample groups per adhesive type (e.g., AVc, AVc + BNC, AVu, AVu + BNC). Mechanical properties were measured for each group. One-way ANOVA (IBM SPSS Statistics, Chicago, IL, USA) was used to compare the effect of BNC addition within each adhesive and surface group, focusing on isolated factor effects. While two-way ANOVA could reveal interaction effects, it was not applied in this study but will be considered in future work. Statistical analysis was performed using Microsoft^®^ Excel 2016 (Microsoft Coorp., Redmond, WA, USA), with significance at *p* < 0.05. Results are presented as mean ± standard deviation.

## 4. Conclusions

In this study, the influence of BNC on the mechanical and adhesive properties of bio-based adhesives formulated from aloe vera and a commercial adhesive was investigated. BNC, derived from a by-product of apple cider vinegar production, was identified as a suitable additive to improve adhesive formulations. Physical characterization of the cardboard substrate confirmed distinct differences between the coated and uncoated surfaces. SEM analysis revealed that effective adhesion on the coated side requires a homogeneous adhesive distribution, whereas the rougher uncoated surface allows for mechanical interlocking, enhancing bond strength when sufficient surface hardness is present.

Due to the relatively high production cost of BNC, a concentration of 2 g per 100 ml adhesive mixture was selected to balance performance benefits with economic feasibility. Despite this limitation, the addition of BNC significantly influenced the mechanical properties of both adhesive systems. In T-peel testing, aloe vera-based adhesives with BNC showed statistically significant increases in maximum peel forces on both coated and uncoated sides. A reduction in work of rupture on the uncoated side indicated decreased ductility, while an increase in work of rupture on the coated side was observed but was not statistically significant, suggesting the need for further investigation with larger sample sizes. Conversely, the effects of BNC addition to commercial adhesives were less pronounced, with decreases in work at maximum force on both surfaces, implying increased brittleness or reduced ductility. Statistically significant differences were confirmed only for the work of rupture on the uncoated side. Shear testing of commercial adhesives revealed failure primarily within the cardboard layers rather than the adhesive interface, indicating strong cohesive properties. For aloe vera adhesives, SEM revealed aggregation on the coated surface, potentially creating weak points, while deeper penetration into the matrix was observed on the uncoated side with minimal residue remaining on the surface.

The study encountered limitations such as cohesive failure during shear testing, suggesting that future evaluations may benefit from adjusting adhesive application areas or using substrates with higher cohesive strength to better assess adhesive performance. While these results demonstrate the promise of BNC-based adhesives as sustainable alternatives to conventional adhesives, their performance was found to depend heavily on the homogeneity of the adhesive mixture. In this study, BNC dispersion was achieved using basic mechanical stirring, which likely limited fiber distribution and contributed to variability in adhesive properties. To address this, future work should explore advanced dispersion techniques such as ultrasonication or high-shear mixing to improve homogeneity and reduce batch-to-batch variability. Additionally, formulation optimization through testing multiple BNC concentrations and potential additives will be essential to balance performance, cost, and processability.

The long-term stability and environmental durability of aloe vera-based adhesives remain important considerations, especially given their hydrophilic nature and sensitivity to humidity, temperature, and microbial exposure. Future studies should incorporate degradation and shelf-life analyses to assess durability and environmental resistance, critical for practical packaging applications, particularly in the food and biodegradable packaging sectors. However, economic viability and scalability analyses are necessary to support the transition of BNC-based adhesives from laboratory research to industrial implementation. This study establishes a correlation between cardboard surface properties, adhesive behavior, and mechanical joint performance, providing valuable insights for the further development of bio-based adhesives. With continued refinement and comprehensive evaluation, BNC-enhanced adhesives have the potential to match or surpass traditional adhesives, while offering significant environmental compatibility and recyclability advantages.

## Figures and Tables

**Table 1:** Results of the basic properties of cardboard (grammage, thickness, density and specific volume of sample cardboard (presented are mean values with standard deviation and no ANOVA analysis).

Properties	Cardboard Sample
Grammage [g/m^2^]	250 ± 1.5
Thickness [mm]	0.45 ± 0.03
Density [g/m^2^]	1.00 ± 0.01
Specific volume [cm^3^/g]	±0.0002

**Table 2:** Results with standard deviation of the porosity, smoothness and Cobb values of the cardboard sample, without adhesives.

Sample	Porosity [mL/min]	Smoothness [mL/min]	Cobb Value [g/m^2^]
Cardboard–uncoated side	51.2 ± 2.9	387.01 ± 2.56	21.00 ± 0.07
Cardboard–coated side	<5	37.25 ± 1.69	16.9 ± 0.55

**Table 3:** *p*-values obtained from the one-way ANOVA analysis for each parameter (porosity, smoothness, and Cobb value) comparing the two groups: uncoated vs. coated side of the cardboard.

Parameter	*p*-Value	Interpretation
Porosity	<0.001	Statistically significant difference
Smoothness	<0.001	Statistically significant difference
Cobb value	<0.01	Statistically significant difference

As all *p*-values are below the 0.05 threshold, it was confirmed that the differences between coated and uncoated sides are statistically significant for all measured parameters. These findings further support the effectiveness of the coating in altering surface properties.

**Table 4:** Results of measured viscosities of the adhesives at 20 °C.

Sample Mixture	Adhesive – Sample Name	Viscosity at 20 °C [Pa∙s]
Aloe vera	AV	10.0
Aloe vera + BNC	AV + BNC	16.2
Commercial adhesive	CA	19.9
Commercial adhesive + BNC	CA + BNC	24.7

**Table 5:** Results of aloe vera with and without BNC additive, average maximum force and work of rupture until maximum force achieved.

Properties	Sample			
	AVc	AVc + BNC	AVu	AVu + BNC
Force [N]	3.35	5.24	0.77	1.19
Standard deviation	0.50	1.03	0.26	0.31
CV [%]	15.03	19.71	33.24	26.02
Work of rupture [N∙mm]	47.71	52.95	8.00	5.30
Standard deviation	14.97	27.24	3.86	1.01
CV [%]	31.39	52.18	48.26	18.97

**Table 6:** One-way ANOVA *p*-values for mechanical properties (force and work of rupture) of aloe vera samples with and without BNC additive.

Parameter	*p*-Value	Interpretation
Force [N]	<0.001	Statistically significant difference
Work of rupture [N∙mm]	<0.001	Statistically significant difference

**Table 7:** Results of commercial adhesive with and without BNC additive, average maximum force and work of rupture until maximum force achieved.

Properties	Sample			
	CAc	CAc + BNC	CAu	CAu + BNC
Force [N]	9.78	11.04	9.46	10.56
Standard deviation	1.99	2.62	2.38	2.89
CV [%]	20.39	23.73	25.13	27.36
Work of rupture [N∙mm]	240.53	158.53	42.66	21.78
Standard deviation	102.73	73.77	5.87	10.55
CV [%]	42.71	46.56	14.34	48.52

**Table 8:** One-way ANOVA *p*-values for mechanical properties (force and work of rupture) of commercial adhesive samples with and without BNC additive, applied to coated and uncoated cardboard surfaces.

Parameter	*p*-Value	Interpretation
Force [N]	0.52	No statistically significant difference
Work of rupture [N∙mm]	<0.001	Statistically significant difference

One-way ANOVA analysis showed that there were no significant differences in the force required for rupture (*p* > 0.05) among the coated and uncoated commercial adhesive samples with or without BNC. However, the work of rupture was significantly different (*p* < 0.01) between groups, indicating that the coating and BNC addition affect the toughness or energy absorption capacity of the adhesive films.

**Table 9:** Preparation of adhesive mixtures, mixing times and speed.

Adhesive and Its Components	Sample Name	Mixing Time [min]	Mixing Speed [rpm]
Pure aloe vera	AV	/	/
Aloe vera + BNC	AV + BNC	15	800
Pure commercial adhesive	CA	/	/
Commercial adhesive + BNC	CA + BNC	15	800

**Table 10:** Classification of adhesive samples based on application and bonding surface of the carton and BNC concentration.

Base Component	Sample Name	Bonding Side of the Carton	Amount of Added BNC
Aloe vera	AVc	Coated	/
Aloe vera	AVu	Uncoated	/
Aloe vera + BNC	AVc + BNC	Coated	2 g into 100 mL aloe vera
Aloe vera + BNC	AVru+ BNC	Uncoated	2 g into 100 mL aloe vera
Commercial adhesive	CAc	Coated	/
Commercial adhesive	CAu	Uncoated	/
Commercial adhesive + BNC	CAc + BNC	Coated	2 g into 100 mL commercial adhesive
Commercial adhesive + BNC	CAu + BNC	Uncoated	2 g into 100 mL commercial adhesive

## Data Availability

No new data were created.
